# Genetic diversity and selection of three nuclear genes in *Schistosoma japonicum* populations

**DOI:** 10.1186/s13071-017-2033-8

**Published:** 2017-02-17

**Authors:** Yaqi Li, Mingbo Yin, Qunfeng Wu, Donald P. McManus, David Blair, Hongyan Li, Bin Xu, Xiaojin Mo, Zheng Feng, Wei Hu

**Affiliations:** 1 School of Life Science, Fudan University, Songhu Road 2005, Shanghai, China; 2 MOE Key Laboratory for Biodiversity Science and Ecological Engineering, School of Life Science, Fudan University, Songhu Road 2005, Shanghai, China; 3 QIMR Berghofer Medical Research Institute, 300 Herston Road, Brisbane, QLD 4029 Australia; 4 College of Science and Engineering, James Cook University, Townsville, QLD 4811 Australia; 5 National Institute of Parasitic Diseases, Chinese Center for Disease Control and Prevention, 207 Rui Jin Er Road, Shanghai, 200025 China

**Keywords:** *Schistosoma japonicum*, Nuclear genes, Genetic diversity, Natural selection, Tegument-associated antigen

## Abstract

**Background:**

The blood fluke, *Schistosoma japonicum* still causes severe disease in China, the Philippines and Indonesia. Although there have been some studies the molecular epidemiology of this persistent and harmful parasite, few have explored the possibility and implications of selection in *S. japonicum* populations.

**Methods:**

We analyzed diversity and looked for evidence of selection at three nuclear genes (*SjIpp2*, *SjFabp* and *SjT22.6*) in 13 *S. japonicum* populations.

**Results:**

*SjT22.6* was found to exhibit high nucleotide diversity and was under positive selection in the mountainous region of mainland China. As a tegumental protein, its secondary and tertiary structure differed between *S. japonicum* strains from the mountainous and lakes regions. In contrast, *SjIpp2* and *SjFabp* had relatively low levels of nucleotide diversity and did not show significant departure from neutrality.

**Conclusions:**

As a tegument-associated antigen-encoding gene of *S. japonicum*, *SjT22.6* has high nucleotide diversity and appears to be under positive selection in the mountainous region of mainland China.

**Electronic supplementary material:**

The online version of this article (doi:10.1186/s13071-017-2033-8) contains supplementary material, which is available to authorized users.

## Background

Approximately 207 million people suffer from schistosomiasis [1]. *Schistosoma japonicum* is endemic in the People’s Republic of China [2], the Philippines [3] and parts of Indonesia [4]. In mainland China, this parasite is particularly prevalent in the lake/marshland regions around the River Yangtze and some mountainous regions in southwest China [5]. Strenuous control efforts during the last five decades have greatly reduced the infection levels and sizes of endemic areas [6]. However, ecosystem changes caused by environmental deterioration and the construction of new infrastructure projects, such as the Three Gorges Dam, contributed to the resurgence of schistosomiasis in the early 21st century [7]. Given the great need for prevention and control of this disease, a thorough understanding of the evolutionary history and population genetic structure of *S. japonicum* is urgently required [8, 9].

Different types of molecular markers have been applied to investigate the genetic variability of *S. japonicum* populations, such as restriction fragment length polymorphism [10], isoenzymes [11], random amplified polymorphic DNA [12], mitochondrial DNA sequences [13, 14] and microsatellites [8, 15]. Recent phylogenetic analyses showed that *S. japonicum* populations in the middle and lower reaches of the River Yangtze are well differentiated from those in the mountainous areas of western China [16–18]. However, so far no study has used specific nuclear genes as molecular markers to assess the effect of selection among Chinese *S. japonicum* populations.

Nuclear genes can be sensitive for addressing questions about genetic variation and in tracing genetic bottlenecks and identifying selection [19]. Liu et al. [20] proposed that three nuclear genes in *S. japonicum* might be under positive selection, including those which encode a protein phosphatase inhibitor 2 (*SjIpp2*), a fatty acid-binding protein (*SjFabp*) and a tegument-associated antigen (*SjT22.6*). The first of these, SjIpp2, likely stops, prevents or reduces the activity of a protein phosphatase [21]. SjFabp belongs to a family of lipid-binding proteins [22] and SjT22.6 is a tegumental surface membrane-anchored calcium-binding antigen, which belongs to a family consisting of platyhelminth tegument-specific proteins [23].

In the present study, we analyzed gene sequences in individual *S. japonicum* worms for *SjIpp2, SjFabp* and *SjT22.6* from 13 populations, including nine locations across mainland China (covering the lakes region and mountainous region) and four locations elsewhere in Asia (Taiwan, Indonesia, Japan and the Philippines). First, we analyzed the diversity of these genes in *S. japonicum* populations. Then we looked for evidence of positive selective pressure acting on these genes as predicted by our previous study [20]. Finally, we used bioinformatics tools to predict whether the protein structure changed under positive selection.

## Methods

### Sample collection

Adult individuals of *S. japonicum* were obtained from 13 locations, including nine from mainland China and four from elsewhere in Asia (Table 1). In mainland China, the sampling was carried out in the lakes region (Guichi and Tongling City in Anhui Province, Shashi City in Hubei Province, Yueyang and Changde City in Hunan Province, Duchang and Nanchang City in Jiangxi Province) and the mountainous region (Eryuan County in Yunnan Province and Xichang City in Sichuan Province). Infected snails (*Oncomelania hupensis*) were collected from each locality in mainland China then transported to the laboratory of NIPD, China CDC, Shanghai. Collection information concerning the infected snails was provided in [24]. In brief, numbers of infected snails from seven localities were as follows: Guichi, 7; Tongling, 10; Shashi, 30; Yueyang, 29; Changde, 21; Duchang, 11; Xichang, 15. Numbers of infected snails from Nanchang and Eryuan are not available. Cercariae were released from pooled infected snails from each locality and used to infect laboratory-raised rabbits. Forty-five days after infection, the adult schistosomes were perfused from the mesenteric veins of infected rabbits and washed in saline, then preserved in 95% (v/v) ethanol at 4 °C. Four other locations from which samples were obtained were Chinese Taiwan (Changhua), Indonesia (Lake Lindu, Sulawesi), Japan (Yamanashi) and the Philippines (Leyte). The lyophilized adult worms from Indonesia and Taiwan were provided by Dr. John Cross, Uniformed Services University of the Health Sciences, Bethesda, USA. The adult worms from Japan (Kofu) were gifted by Dr. Hiroshi Yamasaki, National Institute of Infectious Diseases, Tokyo, Japan. The *S. japonicum* individuals from the Philippines were taken into culture originally in 1969 by Dr. Scholice. The material sent to us consisted of lyophilized adult worms, which was provided by Dr. John Bruce, Centre for Tropical Diseases, University of Lowell, USA.

**Table 1:** Genetic polymorphisms and natural selection of three nuclear genes in *Schistosoma japonicum*

Gene	Region	*N*	Genome sequence						Coding region only						
			L (bp)	H	π	θ^w^	Tajima’s D	Fu’s Fs	L (bp)	H	π	θ^w^	*dN/dS*	Tajima’s D	Fu’s Fs
*SjIpp2*	Lakes^a^	26	992	13	0.005	0.005	0.125	-2.152	594	5	0.003	0.003	0.078	0.052	0.457
	Mountainous^b^	6		6	0.008	0.007	0.405	-1.427		4	0.006	0.005	0.092	0.508	0.426
	TW^c^	4		2	0.003	0.003	-0.797	2.598		2	0.004	0.005	0.000	-0.797	2.598
	IN^c^	4		3	0.004	0.003	1.168	1.031		2	0.005	0.004	0.000	2.080	2.719
	JP^c^	2		2	0.004	0.004	nc	nc		2	0.007	0.007	0.000	nc	nc
	PH^c^	3		2	0.003	0.003	nc	nc		2	0.005	0.005	0.000	nc	nc
	Total	45		21	0.006	0.007	–	–		10	0.004	0.004	0.085	–	–
*SjFabp*	Lakes^a^	33	466	6	0.002	0.003	-0.927	-2.199	304	3	0.000	0.002	0.000	-1.502	-2.477
	Mountainous^b^	10		3	0.001	0.002	-0.184	-0.272		2	0.001	0.001	nc	-1.112	-0.339
	TW^c^	4		1	0.000	0.000	nc	nc		1	0.000	0.000	nc	nc	nc
	IN^c^	5		1	0.000	0.000	nc	nc		1	0.000	0.000	nc	nc	nc
	JP^c^	5		1	0.000	0.000	nc	nc		1	0.000	0.000	nc	nc	nc
	PH^c^	2		1	0.000	0.000	nc	nc		1	0.000	0.000	nc	nc	nc
	Total	59		7	0.002	0.003	–	–		4	0.000	0.002	nc	–	–
*SjT22.6*	Lakes^a^	33	446	10	0.005	0.007	-0.726	-2.308	342	7	0.004	0.004	0.429	-0.377	-1.733
	Mountainous^b^	28		9	0.030	0.016	3.368^***^	6.298^**^		6	0.025	0.013	2.000	3.227^***^	7.259^**^
	TW^c^	4		1	0.000	0.000	nc	nc		1	0.000	0.000	nc	nc	nc
	IN^c^	5		1	0.000	0.000	nc	nc		1	0.000	0.000	nc	nc	nc
	Total	70		17	0.022	0.015	–	–		10	0.018	0.013	1.156	–	–

*Abbreviations*: *N* the number of individuals, *L* length, *H* the number of haplotypes; π, nucleotide diversity, *θ* ^*w*^ the Watterson estimator, *dN* the rates of nonsynonymous substitutions, *dS* the rates of synonymous substitutions, *nc* cannot be calculated

**P < 0.01; ***P < 0.001

^a^Lakes region: AHGC, Guichi, Anhui; AHTL, Tongling, Anhui; HBSS, Shashi, Hubei; HNCD, Changde, Hunan; HNYY, Yueyang, Hunan; JXDC, Duchang, Jiangxi; JXNC, Nanchang, Jiangxi

^b^Mountainous region: SCXC, Xichang, Sichuan; YNEY, Eryuan, Yunnan

^c^Other localities in Asia: TW, Taiwan, China; IN, Indonesia; JP, Japan; PH, the Philippines

Genomic DNA was extracted from each adult worm using the DNeasy Blood & Tissue Kit and Animal Tissues (Spin-Column) protocol from Qiagen (Hilden, Germany), and stored at -20 °C [15, 24]. For most locations, 2–5 individual worms were randomly selected for genetic analysis. However, following initial analyses, 28 additional individual worms were sampled from the mountainous region to examine genetic variation and natural selection in *SjT22.6*.

### Gene amplification and sequencing

To obtain the sequences of the complete gene, we first extracted mRNA sequences of the three targeted gene fragments from NCBI (as only mRNA sequences of these target genes were deposited there): *SjIpp2* (AY815218.1), *SjFabp* (EZ000092.2) and *SjT22.6* (AY813797.1). Using these, we then obtained the complete gene sequences by local Blast searches against the *S. japonicum* genome sequence database [25], enabling us to design primers to amplify and sequence the entire region coding for the mRNA.

The complete mRNA sequence of *SjIpp2* was obtained from NCBI (AY815218.1, 992 bp). The coding regions for this gene are composed of two exons, which were amplified separately from the *S. japonicum* genome DNA using two PCR reactions (Additional file 1: Figure S1a). We analyzed the full length of the gene (sites 1–992) and the coding region (sites 23–616) separately. The complete mRNA sequence of *SjFabp* was obtained from NCBI (EZ000092.2, 699 bp). Only a portion of the coding region and non-coding region of this gene (sites 210–675) could be successfully amplified (Additional file 1: Figure S1b). We analyzed the genome sequence (sites 210 to 675) and the partial coding region (sites 210–513) of this fragment. The complete mRNA sequence of *SjT22.6* was obtained from NCBI (AY813797.1, 887 bp). Again, only a portion of the coding region and non-coding region of this fragment (sites 283–815) was successfully amplified using PCR (Additional file 1: Figure S1c). We analyzed the genome sequence (sites 283–728) and the partial coding region (sites 283–624) of this fragment. The primers used in the present study were shown in Additional file 2: Table S1.

Each DNA fragment was amplified in a 20 μl PCR reaction, containing 1 μl of each primer at a final concentration of 10 μM, 2 μl genomic DNA from an individual worm and 10 μl 2× Taq PCR Master Mix (TaKaRa, Japan). For PCR amplification, templates were denatured at 94 °C for 5 min, followed by 35 cycles with denaturation at 94 °C for 30 s, annealing for 45 s at 51 °C for *SjIpp2*-1, 54 °C for *SjIpp2*-2, 55 °C for *SjFabp*, 46 °C for *SjT22.6* and extension at 72 °C (40s for *SjIpp2*-1, 60 s for *SjIpp2*-2, 100 s for *SjFabp* and 40 s for *SjT22.6*), ending with a final extension at 72 °C for 10 min. All the PCR products were examined using agarose gel electrophoresis (1% w/v) to verify amplification efficiency. The PCR products were sequenced by an ABI 3730 DNA Analyzer (BGI, Shanghai, China) using the PCR primers from both directions. All the sequences were submitted to GenBank under accession numbers KY494243–KY494287 for *SjIpp2*, KY494288–KY494346 for *SjFabp* and KY494347–KY494416 for *SjT22.6*.

### Genetic diversity

The sequences were assembled manually using Vector NTI [26] and aligned using the ClustalW algorithm [27] in MEGA v. 5.10 [28]. The numbers of haplotypes (H), nucleotide diversity (π) and the Watterson estimator (θ^w^) were obtained using DnaSP Version 5 [29]. The Watterson estimator is a method for describing the genetic diversity in a population, which is estimated by counting the number of polymorphic sites. It is a measure of the “population mutation rate” (the product of the effective population size and the neutral mutation rate) from the observed nucleotide diversity of a population [30].

### Haplotype network and selection

The haplotype networks were constructed using HapView [31] to visualize phylogenies. Rates of nonsynonymous substitutions (*dN*) and synonymous substitutions (*dS*) were obtained and Tajima’s D test and Fu’s Fs test were implemented using DnaSP Version 5. A ratio of nonsynonymous to synonymous divergence (*dN/dS*) higher than 1 was considered as evidence of positive selection [32]. Furthermore, Tajima’s D test and Fu’s Fs test were applied to test for departure from neutral evolution. Tajima’s D test is calculated according to the difference between segregating sites and the average of nucleotide differences [33], and Fu’s Fs test compares the number of haplotypes observed with the expected number of haplotypes in a random sample [34].

### Protein structure

Inferred amino acid sequences were aligned using the ClustalW algorithm in MEGA 5.10. Protein models were predicted by Phyre2 [35], and the energy minimization of the model was performed using YAMASA [36]. Secondary structures were extracted from the predicted model by ProFunc [37]. Protein-ligand binding sites were predicted using COFACTOR [38–40]. Structural visualization and editing were done using PyMol software (http://www.pymol.org).

## Results

### Genetic diversity

In total, 45 individual *S. japonicum* adult worms were successfully sequenced for *SjIpp2*, 59 for *SjFabp* and 70 for *SjT22.6*. Among these, 21 unique haplotypes were detected for *SjIpp2*, 7 for *SjFabp* and 17 for *SjT22.6*; 10, 4 and 10 unique haplotypes were obtained for the coding regions of the three genes, respectively (Table 1 and Additional file 3: Table S2).

Overall, the level of nucleotide diversity within *S. japonicum* populations was low for *SjIpp2* (π = 0.006) and *SjFabp* (π = 0.002). In contrast, the nucleotide diversity for *SjT22.6* (π = 0.022) was approximately 4-fold higher than that for *SjIpp2* and 10-fold higher than that for *SjFabp*. Furthermore, the Watterson estimator for this gene (θ^w^ = 0.015) was 2 times higher than *SjIpp2* (θ^w^ = 0.007) and 5 times higher than *SjFabp* (θ^w^ = 0.003). Interestingly, for *SjT22.6*, the nucleotide diversity of *S. japonicum* populations from the mountainous region (π = 0.030) was approximately six times greater than those from the lakes region (π = 0.005). Outside mainland China, the *SjT22.6* sequences from Chinese Taiwan or Indonesia worms were identical and this gene could not be amplified for individuals from Japan and the Philippines.

### Haplotype network

**Fig. 1:**
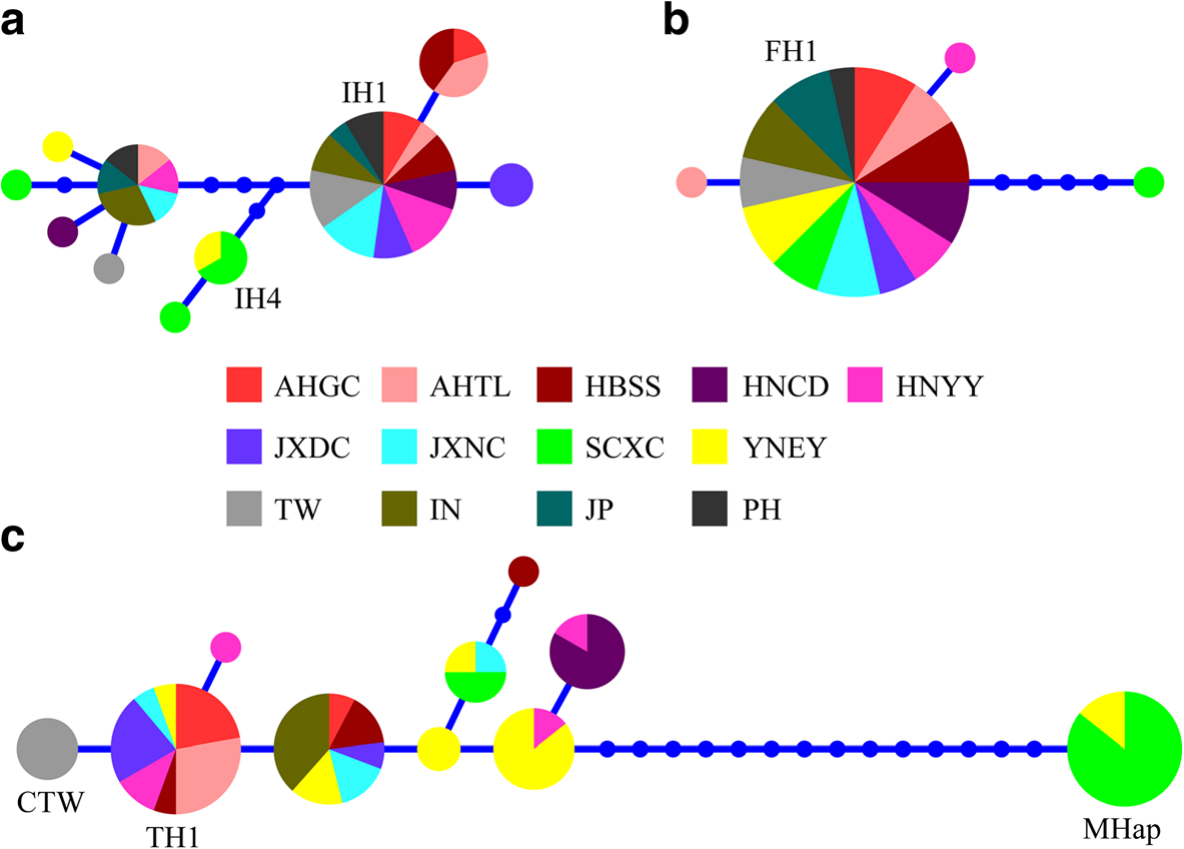
Haplotype networks for *Schistosoma japonicum* based on the coding region of *SjIpp2* (**a**), *SjFabp* (**b**) and *SjT22.6* (**c**). Each color represents a locality. The distance between two haplotypes corresponds to the numbers of substitutions. Abbreviations of the geographical localities are shown in Table 1

The most abundant haplotype of the *SjIpp2* coding region (IH1; 23 out of 45) was shared by the 11 locations in the lakes region of mainland China and elsewhere (Fig. 1a), but was not found in mountainous regions of the mainland. For the *SjFabp* coding region, almost all of the *S. japonicum* individuals shared a single haplotype (FH1; 56 out of 59, Fig. 1b), regardless of geographical origin. For the *SjT22.6* coding region, the most abundant haplotype (TH1; 18 out of 70) was shared by seven locations in mainland China, including Sichuan; the second most abundant haplotype (MHap; 14 out of 70) was obviously very distinct from all others (Fig. 1c), and found only in worms from mountainous regions (Sichuan and Yunnan).

The network of the *SjIpp2* coding region sequences showed that a single haplotype (IH4, Fig. 1a), was common only at the two mountainous localities in China. Similarly, one of the haplotypes for the *SjT22.6* coding region (MHap, Fig. 1c) was only found among worms from the mountainous regions, where it was common. Otherwise there was no obvious geographical distinction between mainland China and elsewhere. However, in the network of the *SjT22.6* coding region, all the Chinese Taiwan individuals exhibited a single haplotype (CTW, Fig. 1c), distinct from others. The networks generated from genome sequences (coding + non-coding) (Additional file 4: Figure S2) were consistent with those inferred from coding region sequences only (Fig. 1).

### Selection

The *dN/dS* values were less than one among all the populations for *SjIpp2* or *SjFabp*, suggesting both of them were under negative or purifying selection. However, The Tajima’s D and Fu’s Fs tests did not show significant departure from neutrality. This was also observed in *S. japonicum* populations from the lakes region for *SjT22.6*. However, for this gene, the *dN/dS* radio was two for the alignment of *S. japonicum* individuals from mountainous regions, suggesting positive selection. This was further supported by the Tajima’s test (D = 3.227, *P* < 0.001) and Fu’s test (Fs = 7.259, *P* < 0.01) (Table 1).

### Protein structure prediction

**Fig. 2:**
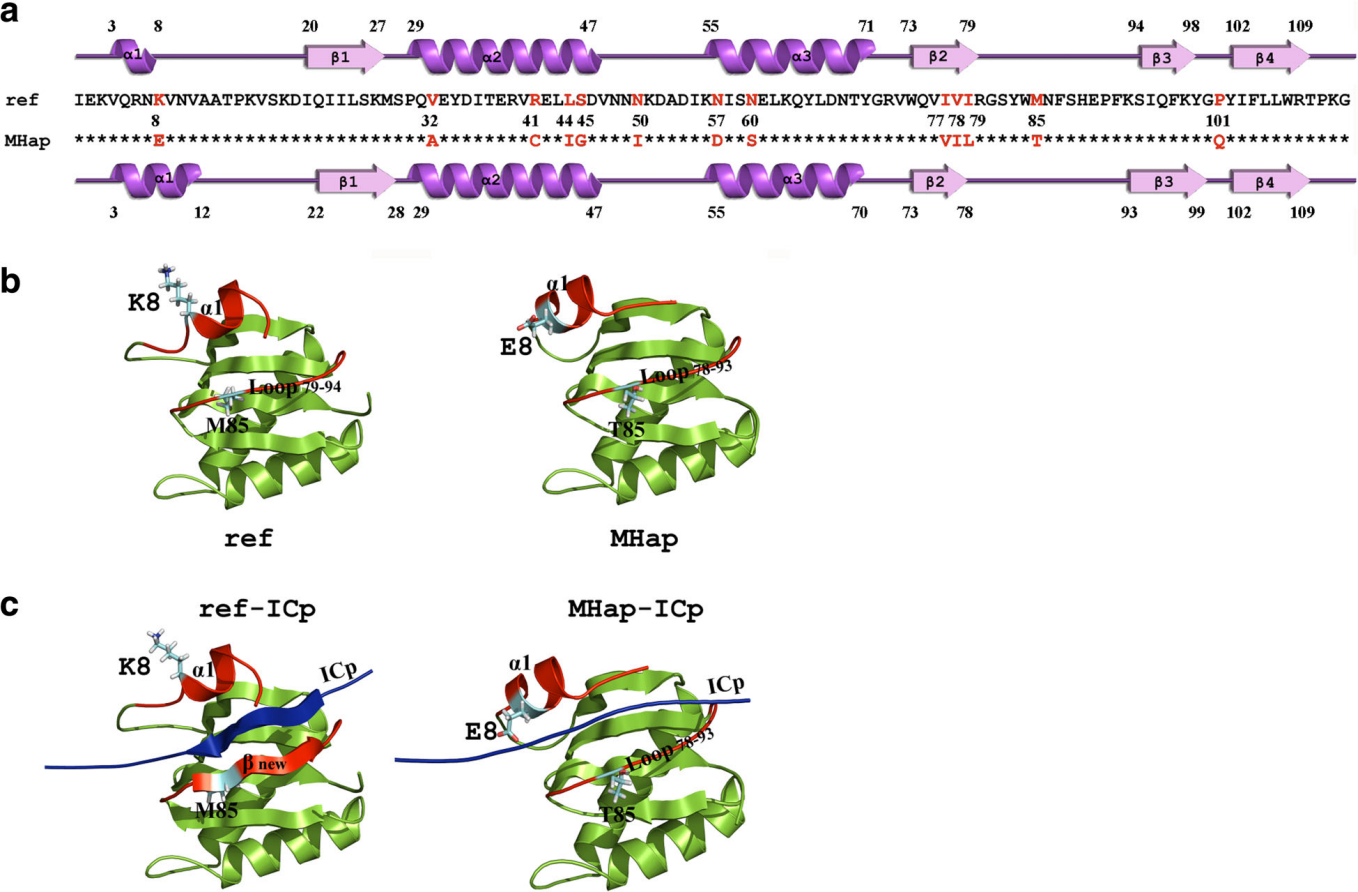
Protein structure prediction based on the coding region of *SjT22.6* for the main mountainous region haplotype and the reference sequence. **a** Secondary structure. **b** Predicted tertiary structure. **c** The binding affinity of *SjT22.6* with the potential peptide (ICp) for MHap and the reference sequence

Protein sequence analysis of *SjT22.6* showed that the variations mainly occurred between MHap and other haplotypes (Additional file 5: Figure S3), so further comparison between MHap and the reference sequence (from lake region, GenBank ID: AAW25529.1) was conducted. The secondary structure of the two sequences differed at 13 amino acid sites. Three α-helixes (α1, α2, α3) and four β-shifts (β1, β2, β3, β4) were detected in both of the sequences (Fig. 2a). The length of α1 inferred from the reference sequence was shorter than in MHap, leading to differences in tertiary structures (Fig. 2b).

Functional predictions showed that *SjT22.6* was homologous to dynein light chain 1, a member of the dynein superfamily. The major functional identification region encompassed the first 20 amino acids (Additional file 6: Table S3) where α1 existed, with one amino acid substitution (K8ref vs E8MHap). The binding site in MHap and the reference sequence, characterized as the conserved motif KXTQT [41], differed in binding strength with dynein intermediate chain (ICp) [42]. Obviously, ICp interacted more powerfully with the reference protein than with MHap (Fig. 2c), indicating functional divergence between these two proteins.

## Discussion

In this study, we found that the nuclear gene *SjT22.6* of *S. japonicum* was under positive selection in the mountainous area of mainland China. Furthermore, the secondary and tertiary structures of the protein encoded by *SjT22.6* differed in some *S. japonicum* individuals from the mountainous region, when compared with those from the lakes region and elsewhere in Asia.

A high level of nucleotide diversity was detected in *SjT22.6*, a gene encoding a tegument-associated antigen [43]. Such high diversity has also been found in other membrane proteins of *S. japonicum* [44, 45]. In general, high levels of polymorphism in proteins are due to recombination events and favored by positive selection in which selective forces, such as immune responses and drugs, drive the accumulation of mutations and maintain diversity in the population [46]. Many previous studies have indicated that genes involved in infection or maintaining important functions can exhibit particularly high mutation rates [47–49]. Thus, SjT22.6, as an immune response target [43], has tended to evolve more rapidly. The accumulated mutations may alter the protein sequence and help the worms to evade host immune attack. Interestingly, in the present study, *SjT22.6* could not be amplified in *S. japonicum* strains from Japan and the Philippines. This was unexpected because the blood flukes in these two countries both have ancestors from mainland China [16]. This lack of amplification might be due to mutations occurring in the primer regions following the arrival of the parasites in Japan and the Philippines [50].

In this study, positive selection of *SjT22.6* has occurred in the mountainous region of mainland China, but not in other regions. Several research groups have shown strong differentiation between *S. japonicum* populations in mountainous regions and those in the lakes area of the middle and lower Yangtze [16–18]. Among the selection pressures that might specifically impinge upon the blood flukes in the mountainous regions are the differences in intermediate and final hosts. While only speculation at this stage, adaptations to new hosts, and especially to a different subspecies of snail host, may have left the signature of selection in the population. Subspecies of *Oncomelania hupensis* have substantial genetic and morphological differences among different endemic areas of schistosomiasis japonica [51–53]. Therefore, substantial genetic change might accompany a geographical and host shift. The major definitive hosts of *S. japonicum* in the lakes region of mainland China are bovines and humans [54], while in the mountainous region, in addition to bovines and humans, there is a wide range of additional hosts, such as rodents and dogs [55].

As a platyhelminth-specific calcium-binding antigen, SjT22.6 is believed to have an N-terminal calcium-binding EF-hand domain and a C-terminal dynein light chain-like domain (DLC) [56]. The DLC domains of tegumental proteins are important for normal cellular homeostasis [57]. In *S. mansoni*, they might participate in the transport of vesicles within the tegumental cytoplasm [58] and the shuttling of vesicles into the tegument surface [59], probably within dynein motor complexes. In this study, the first α-helix of the main haplotype of *SjT22.6* in the mountainous region (MHap) was longer and had less binding power than that of the reference sequence. These changes might have potential effects on the cellular homeostasis of *S. japonicum*, which might further influence the host-parasite interaction.

The other two gene fragments, *SjFabp* and *SjIpp2*, had low levels of nucleotide diversity. For *SjFabp*, most individuals shared a single haplotype and the *dN/dS* ratio was less than one. Thus, this gene fragment might be under purifying selection. Furthermore, the networks of these three genes indicated that the Sichuan and Yunnan populations from mountainous areas often shared haplotypes. This might reflect common ancestry [16] rather than gene flow between the two populations, which is rare because of the geographical isolation.

## Conclusions

In conclusion, as a tegument-associated antigen-encoding gene of *S. japonicum*, *SjT22.6* has high nucleotide diversity, appearing to be under positive selection in the mountainous region of mainland China. The protein structure and binding power of the most common *SjT22.6* haplotype in the mountainous region differed from those of the reference sequence and of many haplotypes from elsewhere. In contrast, *SjIpp2* and *SjFabp* had relatively lower diversity and did not show signatures of positive selection. Future research should aim to obtain a comprehensive understanding of the specific function of *SjT22.6*, such as its effect on the interaction between the worm and its hosts. Overall, by using three nuclear genes as genetic markers, the current findings provide valuable insights and fundamental genetic and evolutionary information concerning *S. japonicum*. Further research on these, and particularly on *SjT22.6*, is clearly required to confirm whether these can contribute to new interventions in the fight to eliminate schistosomiasis.

## Additional files

Additional file 1: Figure S1. The locations of the three genes in *S. japonicum* genome for *SjIpp2* (**a**), *SjFabp* (**b**) and *SjT22.6* (**c**). The red rectangles represent the coding regions of the genes. The blue rectangles represent the non-coding regions of these genes. (PDF 225 kb)
Additional file 2: Table S1. Primers sequences for the three genomic fragments. (DOCX 13 kb)
Additional file 3: Table S2. Numbers of observed haplotypes of *S. japonicum* isolates from different locations based on three genomic fragments. (DOCX 15 kb)
Additional file 4: Figure S2. Networks for haplotypes of *S. japonicum* based on three genome fragments with (**a**) *SjIpp2*, (**b**) *SjFabp* and (**c**) *SjT22.6*. Each color represents a locality. The distance between two haplotypes corresponds to the number of substitutions. Abbreviations of the geographical localities are shown in Table 1. (PDF 199 kb)
Additional file 5: Figure S3. Haplotype network for *S. japonicum* based on the amino acid sequence of *SjT22.6*. Each color represents a locality. The distance between two haplotypes corresponded to the number of substitutions. The number represents each mutation site of MHap compared with the reference sequence. Abbreviations of the geographical localities are shown in Table 1. (PDF 195 kb)
Additional file 6: Table S3. The structural alignment of the reference sequence (ref, AAW25529.1) and MHap. (DOCX 13 kb)
