# Supplementary material for: Genetic diversity and selection of three nuclear genes in Schistosoma japonicum populations
Source: Parasit Vectors. 2017 Feb 17;10:87. doi: 10.1186/s13071-017-2033-8 (PMC5316221; doi:10.1186/s13071-017-2033-8)

Additional file 1: Figure S1. The locations of the three genes in *S. japonicum* genome for *SjIpp2* (**a**), *SjFabp* (**b**) and *SjT22.6* (**c**). The red rectangles represent the coding regions of the genes. The blue rectangles represent the non-coding regions of these genes. Primer-1: SjIpp2-1F/R; Primer-2: SjIpp2-2F/R; Primer-3: SjFabp-F/R; Primer-4: SjT22.6-F/R.

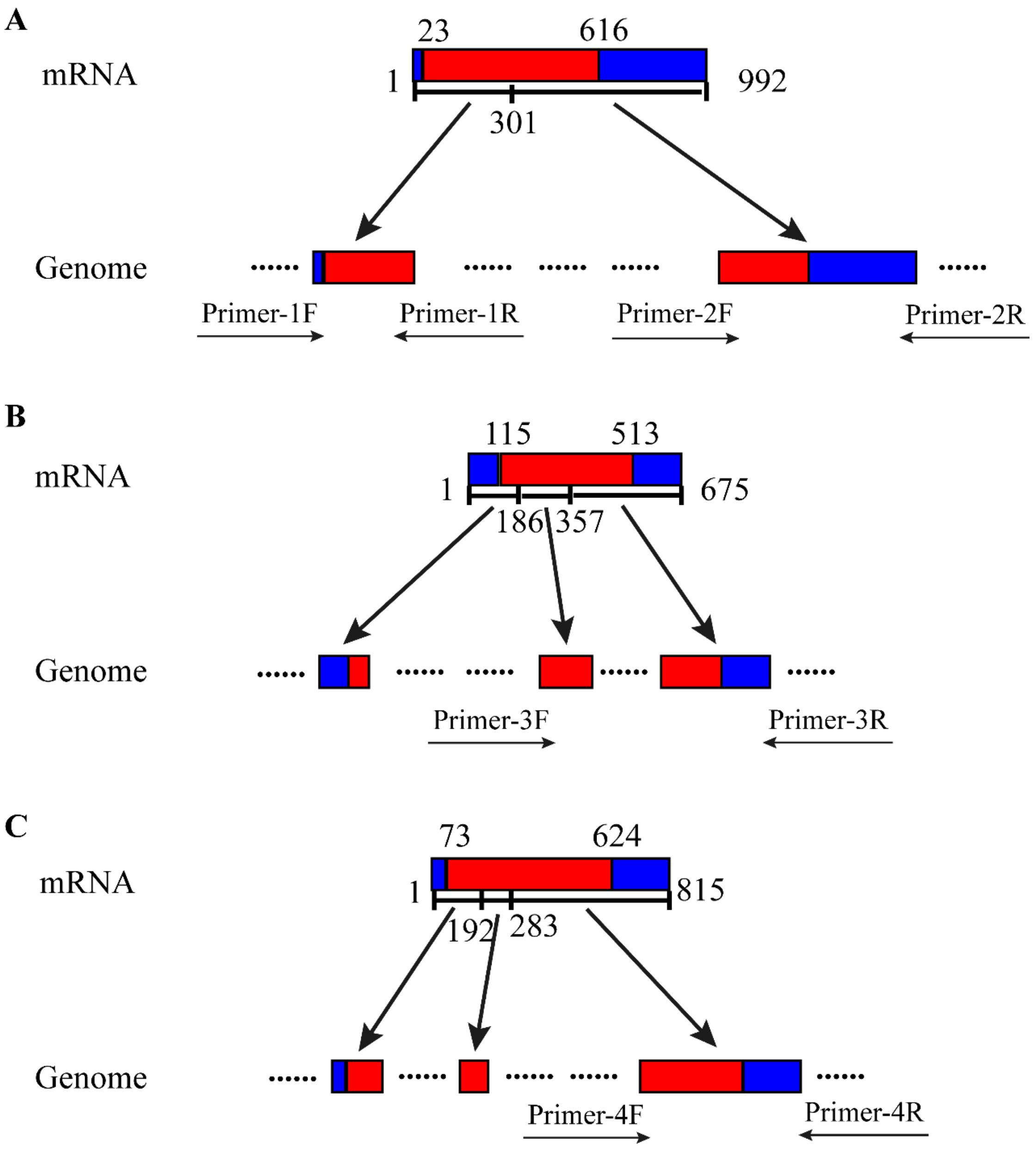

Supplement: Additional file 1: Figure S1. — The locations of the three genes in S. japonicum genome for SjIpp2 (a), SjFabp (b) and SjT22.6 (c). The red rectangles represent the coding regions of the genes. The blue rectangles represent the non-coding regions of these genes. (PDF 225 kb) [file 13071_2017_2033_MOESM1_ESM.pdf]
