# Supplementary material for: Genetic diversity and selection of three nuclear genes in Schistosoma japonicum populations
Source: Parasit Vectors. 2017 Feb 17;10:87. doi: 10.1186/s13071-017-2033-8 (PMC5316221; doi:10.1186/s13071-017-2033-8)
Supplement: Additional file 2: Table S1. — Primers sequences for the three genomic fragments. (DOCX 13 kb) [file 13071_2017_2033_MOESM2_ESM.docx]

**Additional file 2: Table S1.** Primers sequences for the three genomic fragments.

| **Names** | **Primers 5’-3’** |
| --- | --- |
| *SjIpp2*-1-F | TAC GTA GAT GCA AAT CCT AAG AC |
| *SjIpp2*-1-R | AGA CAT TTA AAA ACC ACC CAT A |
| *SjIpp2*-2-F | TTG ACA CAT TGG CAT ATA TCT TA |
| *SjIpp2*-2-R | GAC GTT TGT AAA CTT AAA TGC A |
| *SjFabp*-F | TCG TAC CAA GAA TAG TTC TCC TAA T |
| *SjFabp*-R | ACA TGC ATT CGA AAC CGT AAG |
| *SjT22.6*-F | GTT TTT ATT CTT TAA AAT ATA AAT C |
| *SjT22.6*-R | AGT AAA TCT ATT CTC TAA CAC AAT G |
