# Supplementary material for: Genetic diversity and selection of three nuclear genes in Schistosoma japonicum populations
Source: Parasit Vectors. 2017 Feb 17;10:87. doi: 10.1186/s13071-017-2033-8 (PMC5316221; doi:10.1186/s13071-017-2033-8)
Supplement: Additional file 3: Table S2. — Numbers of observed haplotypes of S. japonicum isolates from different locations based on three genomic fragments. (DOCX 15 kb) [file 13071_2017_2033_MOESM3_ESM.docx]

**Additional file 3: Table S2** Numbers of observed haplotypes of *S. japonicum* isolates from different locations based on three genomic fragments.

| Population | *SjIpp2* Haplotype | *SjFabp* Haplotype | *SjT20.2* Haplotype |
| --- | --- | --- | --- |
| **Genome sequence** | |  |  |
| AHGC | IH1 (2), IH3 (1) | FH1 (3), FH2 (2) | TH1 (4), TH2 (1) |
| AHTL | IH2 (1), IH5 (1), AHTLa (1) | FH1 (2), FH2 (2), AHTLa (1) | TH1 (5) |
| HBSS | IH2 (2) , IH5 (2) | FH1 (4), FH2 (1) | TH1 (1), TH2 (2), HBSSa (1) |
| HNCD | HNCDa (1), HNCDb (1), HNCDc (1) | FH1 (1), FH2 (4) | TH3 (5) |
| HNYY | IH1 (1), IH2 (2), IH3 (1) | FH1 (2), FH2 (1), FH3 (1), HNYYa (1) | TH1 (2), TH3 (1), TH4 (1), HNYYa (1) |
| JXDC | IH1 (1), IH4 (1), JXDCa (1), JXDCb (1) | FH1 (1), JXDCa (2) | TH1 (2), TH2 (1), JXDCa (1), JXDCb (1) |
| JXNC | IH1 (2), IH3 (1), JXNCa (1) | FH1 (3), FH2 (2) | TH1 (1), TH2 (1), TH5 (1), JXNCa (1) |
| SCXC | SCXCa (1), SCXCb (1), SCXCc (1), SCXCd (1) | FH1 (2), FH2 (2), SCXCa (1) | MHap1 (11), MHap2 (2), SCXCa (1) |
| YNEY | YNEYa (1), YNEYb (1) | FH1 (5) | MHap1 (2), MHap2 (1), TH1 (1), TH3 (3), TH5 (2), YNEYa (1), YNEYb (1), YNEYc (1), YNEYd (2) |
| CTW | IH2 (3), CTWa (1) | FH2 (4) | CTW (4) |
| IN | IH1 (2), IH3 (1), INa (1) | FH3 (5) | TH2 (5) |
| JP | IH1 (1), IH3 (1) | FH3 (5) | n.c. |
| PH | IH1 (2), IH3 (1) | FH3 (2) | n.c. |
| **Coding region** | |  |  |
| AHGC | IH1 (2), IH3 (1) | FH1 (5) | TH1 (4), TH2 (1) |
| AHTL | IH1 (1), IH2 (1), IH3 (2) | FH1 (4), AHTLa (1) | TH1 (5) |
| HBSS | IH1 (2), IH3 (2) | FH1 (5) | TH1 (1), TH2 (2), HBSSa (1) |
| HNCD | IH1 (2), HNCDa (1) | FH1 (5) | TH3 (5) |
| HNYY | IH1 (3), IH2 (1) | FH1 (4), HNYYa (1) | TH1 (2), TH3 (1), TH4 (1), HNYYa (1) |
| JXDC | IH1 (2), JXDCa (2) | FH1 (3) | TH1 (4), TH2 (1) |
| JXNC | IH1 (3), IH2 (1) | FH1 (5) | TH1 (1), TH2 (2), TH5 (1) |
| SCXC | IH4 (2), SCXCa (1), SCXCb (1) | FH1 (4), SCXCa (1) | MHap (12), TH5 (2) |
| YNEY | IH4 (1), YNEYa (1) | FH1 (5) | MHap (2), TH1 (1), TH2 (2), TH4 (6), TH5 (1), YNEYa (2) |
| CTW | IH1 (3), CTWa (1) | FH1 (4) | CTW (4) |
| IN | IH1 (2), IH2 (2) | FH1 (5) | TH2 (5) |
| JP | IH1 (1), IH2 (1) | FH1 (5) | n.c. |
| PH | IH1 (2), IH2 (1) | FH1 (2) | n.c. |

IH, different individuals shared the same *SjIpp2* Haplotype; FH, different individuals shared the same *SjFabp* Haplotype; TH, different individuals shared the same *SjT20.2* haplotype; MHap, individuals only from mountainous region (YNEY and SCXC) shared the same *SjT20.2* haplotype. The numbers in parentheses indicate number of individuals possessing the identical sequence per locality
