# Supplementary material for: Genetic diversity and selection of three nuclear genes in Schistosoma japonicum populations
Source: Parasit Vectors. 2017 Feb 17;10:87. doi: 10.1186/s13071-017-2033-8 (PMC5316221; doi:10.1186/s13071-017-2033-8)

Additional file 4: Figure S2. Networks for haplotypes of *S. japonicum* based on three genome fragments with (**a**) *SjIpp2*, (**b**) *SjFabp* and (**c**) *SjT22.6*. Each color represents a locality. The distance between two haplotypes corresponds to the number of substitutions. Abbreviations of the geographical localities are shown in Table 1.

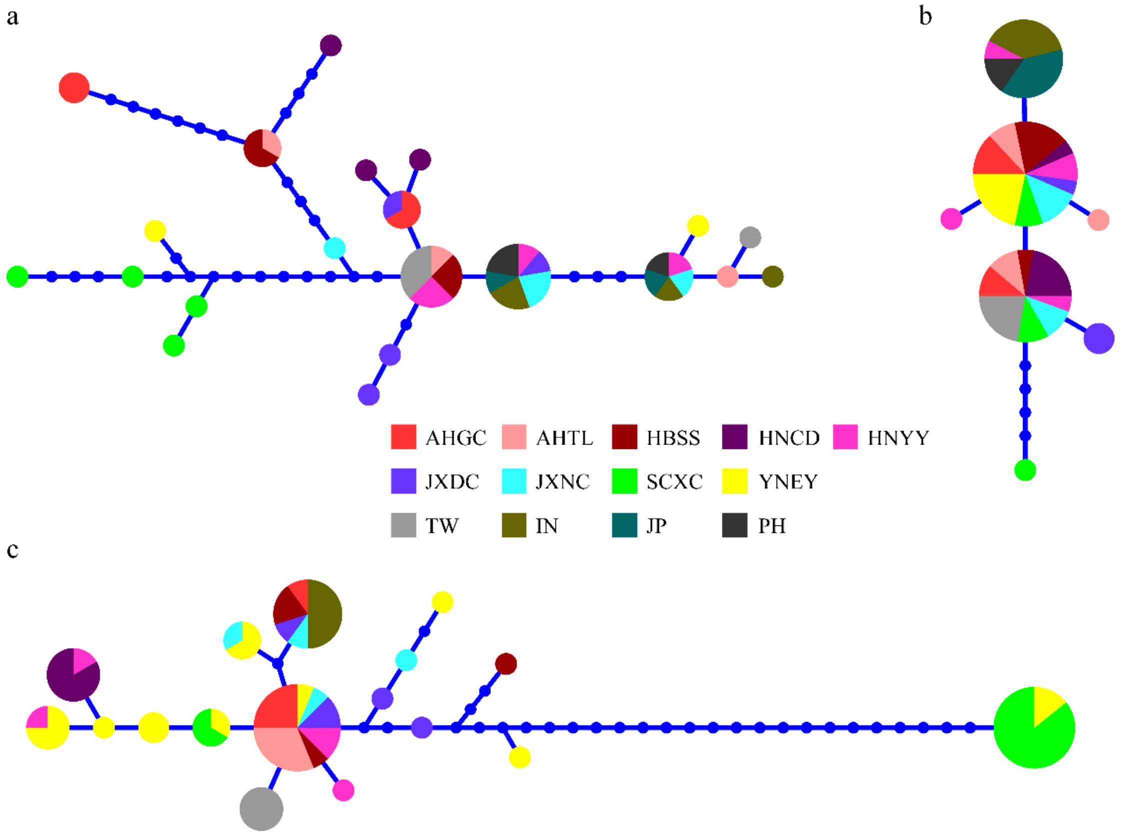

Supplement: Additional file 4: Figure S2. — Networks for haplotypes of S. japonicum based on three genome fragments with (a) SjIpp2, (b) SjFabp and (c) SjT22.6. Each color represents a locality. The distance between two haplotypes corresponds to the number of substitutions. Abbreviations of the geographical localities are shown in Table 1. (PDF 199 kb) [file 13071_2017_2033_MOESM4_ESM.pdf]
