# Supplementary material for: Genetic diversity and selection of three nuclear genes in Schistosoma japonicum populations
Source: Parasit Vectors. 2017 Feb 17;10:87. doi: 10.1186/s13071-017-2033-8 (PMC5316221; doi:10.1186/s13071-017-2033-8)

Additional file 5: Figure S3. Haplotype network for *S. japonicum* based on the amino acid sequence of *SjT22.6*. Each color represents a locality. The distance between two haplotypes corresponded to the number of substitutions. The number represents each mutation site of MHap compared with the reference sequence. Abbreviations of the geographical localities are shown in Table 1.

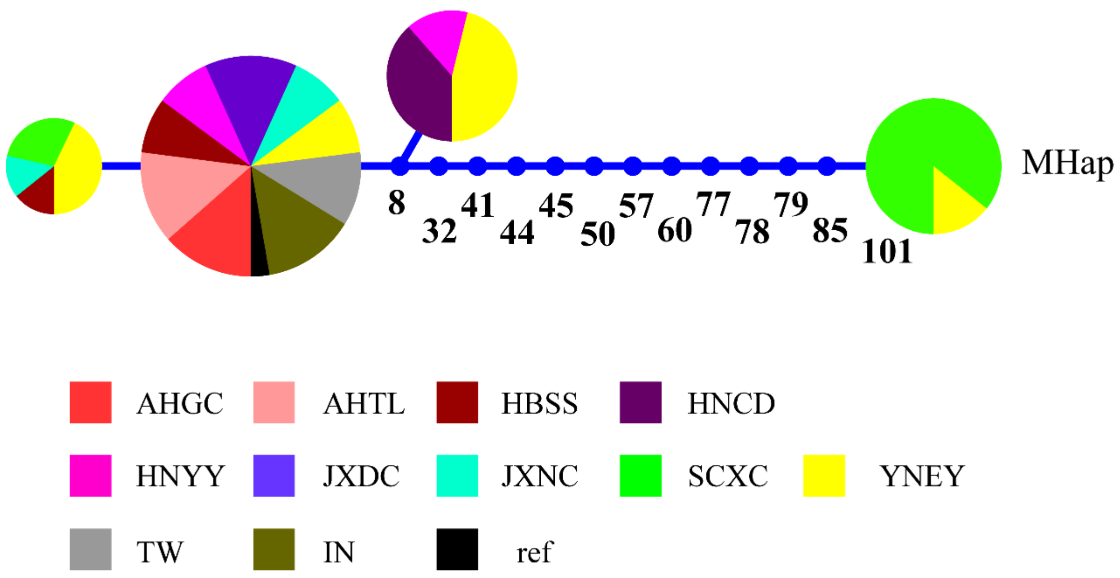

Supplement: Additional file 5: Figure S3. — Haplotype network for S. japonicum based on the amino acid sequence of SjT22.6. Each color represents a locality. The distance between two haplotypes corresponded to the number of substitutions. The number represents each mutation site of MHap compared with the reference sequence. Abbreviations of the geographical localities are shown in Table 1. (PDF 195 kb) [file 13071_2017_2033_MOESM5_ESM.pdf]
