# Supplementary material for: Genetic diversity and selection of three nuclear genes in Schistosoma japonicum populations
Source: Parasit Vectors. 2017 Feb 17;10:87. doi: 10.1186/s13071-017-2033-8 (PMC5316221; doi:10.1186/s13071-017-2033-8)
Supplement: Additional file 6: Table S3. — The structural alignment of the reference sequence (ref, AAW25529.1) and MHap. (DOCX 13 kb) [file 13071_2017_2033_MOESM6_ESM.docx]

**Additional file 6: Table S3.** The structural alignment of the reference sequence (ref,

AAW25529.1) and MHap.

|  | RMSD |
| --- | --- |
| Whole structure of ref and MHap | 3.421 Å |
| Residues 21-112 in ref and MHap | 1.643 Å |
| Residues 1-20 in ref and MHap | 6.464 Å |
